# Design theory to better target public health priorities: An application to Lyme disease in France

**DOI:** 10.3389/fpubh.2022.980086

**Published:** 2022-11-07

**Authors:** Gwenaël Vourc'h, Katherine Un, Elsa Berthet, Pascale Frey-Klett, Pascal Le Masson, Benoit Weil, Olivier Lesens

**Affiliations:** ^1^INRAE, VetAgro Sup, UMR EPIA, Université Clermont Auvergne, Saint Genès Champanelle, France; ^2^INRAE, VetAgro Sup, UMR EPIA, Université de Lyon, Marcy l'Etoile, France; ^3^Centre de Gestion Scientifique, i3 UMR CNRS 9217, MINES ParisTech, PSL Research University, Paris, France; ^4^INRAE, AgroParisTech, UMR SADAPT, Université Paris-Saclay, Paris, France; ^5^USC 1339, Centre d'Etudes Biologiques de Chizé, INRAE, Villiers-en-Bois, France; ^6^UMR 7372 Centre d'Études Biologiques de Chizé, CNRS, Univ. La Rochelle, Beauvoir-sur-Niort, France; ^7^US 1371 Laboratory of Excellence ARBRE, INRAE, Champenoux, France; ^8^Université Clermont Auvergne UMR, CNRS 6023, Laboratoire Microorganismes: Génome Environnement (LMGE), Clermont-Ferrand, France; ^9^Service des maladies infectieuses et tropicales, Centre de référence pour la prise en charge des infections ostéo-articulaires complexes (CRIOA), Centre de Référence pour les Maladies Vectorielles liées aux Tiques (CRMVT), 3IHP, CHU, Clermont-Ferrand, France

**Keywords:** Lyme disease, innovative design, public health prevention, tick-borne diseases, design theories, concept-knowledge, chronic diseases

## Abstract

In the context of complex public health challenges led by interdependent changes such as climate change, biodiversity loss, and resistance to treatment, it is important to mobilize methods that guide us to generate innovative interventions in a context of uncertainty and unknown. Here, we mobilized the concept-knowledge (CK) design theory to identify innovative, cross-sectoral, and cross-disciplinary research and design programs that address the challenges posed by tick-borne Lyme disease in France, which is of growing importance in the French public health and healthcare systems. Within the CK methodological framework, we developed an iterative approach based on literature analysis, expert interviews, analysis of active French research projects, and work with CK experts to contribute to design “an action plan against Lyme disease.” We produced a CK diagram that highlights innovative concepts that could be addressed in research projects. The outcome is discussed within four areas: (i) effectiveness; (ii) environmental sustainability in prevention actions; (iii) the promotion of constructive involvement of citizens in Lyme challenges; and (iv) the development of care protocols for chronic conditions with an unknown diagnosis. Altogether, our analysis questioned the health targets ranging from population to ecosystem, the citizen involvement, and the patient consideration. This means integrating social and ecological science, as well as the multidisciplinary medical patient journey, from the start. CK theory is a promising framework to assist public health professionals in designing programs for complex yet urgent contexts, where research and data collection are still not sufficient to provide clear guidance.

## Introduction

The world is facing many complex and interdependent changes, such as climate change, loss of biodiversity, increased antimicrobial resistance, and higher frequency of outbreak of zoonotic diseases (1–4). Such changes are difficult to fully apprehend since they are both impacted by daily, entrenched human activities as well as environmental entangled factors beyond control by simple measures. The complexity of the issues at stake challenges the nature of public health work, as illustrated by the COVID-19 crisis, which featured uncertain, novel situations, and unprecedented modes of actions. Experts and researchers are urged to develop programs to prevent public health crises.

The current usual framework for building a response to public health crises typically describes a linear path from problem definition, the generation of a set of alternative solutions, the subsequent evaluation of these options, and finally the study and practice of implementation and dissemination. For instance, cost-effectiveness analysis, comparative effectiveness research, and dissemination and implementation sciences have been mobilized (5, 6). Yet, focusing on such approaches entrenches the mind in a set of choices where the objectives, pathways, and options, even if novel, are well identified. They fail to overcome two characteristics that are of particular importance for innovation in complex situations: (i) the automatic use of easily accessible knowledge to solve a problem leading to a cognitive bias called “fixation effect” and constraining the exploration of solutions from a restricted number of categories (7, 8), and (ii) the unknown or ill-characterized aspects regarding the desired objective to reach, the paths to use, the knowledge to mobilize, the organization to be developed, or the actors to involve.

Developing a process to have a systemized, methodic way to generate innovative ideas in a context of uncertainty and unknowns is the core of innovative design. The concept-knowledge (CK) theory is a theory of innovative design (9, 10) that formally distinguishes “knowledge” from “concepts.” Knowledge is considered as ideas and propositions with a logical status (true or false), and concepts as ideas and propositions without logical status (neither true nor false) but that are desirable and not impossible, e.g., “Antibiotics without resistance.” The distinct space between knowledge and concept helps to explicitly and systematically organize the process of exploring innovative ideas. The formalism of CK allows the user to distinguish exploratory pathways based on the spontaneous activation of knowledge (i.e., fixation effects) from exploratory pathways that rely on the activation of less accessible knowledge (outside fixation effects). Once fixation effects are identified, it is possible to develop levers to overcome them and stimulate creativity (11). The diagram structure of concept exploration in CK opens unexpected pathways, by requiring the consideration of contrasting ideas, for instance, a path with Property ≪ P ≫ (e.g., tick bite with *Borrelia* transmission) and a path with Property ≪ non-P ≫ (e.g., tick bite with no *Borrelia* transmission).

The concept-knowledge (CK) theory gave birth to a participatory innovative design method called KCP that has been applied in industrial contexts for a long time [Knowledge-Concepts-Proposals, (12)]. The method is based on several collective workshops that bring a large and heterogenous collective of stakeholders to conduct innovative design reasoning together. It has now a broad range of applications, such as the fostering of cross-disciplinary research (13) or promoting agroecological practices (14) or generating alternative in public decision making (15).

In this article, we describe an application of the concept-knowledge (CK) theory (9, 10) to a healthcare and public health issue in order to generate new research questions and identify innovative paths for public actions. We targeted a complex public health challenge, the issue of Lyme disease (LD), a tick-borne infection caused by pathogenic bacteria of the *Borrelia burgdorferi* group. It is currently the most prevalent vector-borne zoonosis in the temperate regions of the Northern hemisphere (16, 17) and imposes a high economic burden (18, 19).

Complexity in LD resides in the uncertainty, unknown, and diverging knowledge as well as in the diversity of stakeholders involved in the prevention, diagnosis, and treatment, all of which put a growing burden on the French public health and healthcare systems (20). Approaches to control tick populations are diverse and include acaricides, landscape management, and control of hosts that feed reproductive adult ticks (21). No human vaccines are yet available (22, 23). LD typically evolves in three stages (24): early localized stage, early disseminated stage, and late stage. The diagnosis is based on the combination of the patient's exposure to risk, clinical symptoms, and serological evidence (25). The infection is treated with a 2- to 4-week course of antibiotics. Post-treatment Lyme disease syndrome (PTLDS) affects an estimated 10% of patients after they have been diagnosed and treated for LD (26, 27). Uncertainties and controversies about LD diagnosis and management have led to alternative theories that promote alternative medical care based more on practical experience than scientific proof. In this context, the chronic stage of the disease has been described and named “Chronic Lyme disease” (CLD). The symptoms included are unspecific (28) and occur at the same rate as in patients who were diagnosed with or self-identify as having CLD than in the average population (29, 30). Some healthcare practitioners prescribe long-term antibiotic courses with no proven efficacy (29).

In 2016, in the face of the disease's growing importance and supported by patient association action and press coverage, the French government launched a national action plan to improve the prevention, diagnosis, and treatment of LD and other tick-borne diseases (31). A first draft of the plan was elaborated by the French General Directorate for Health. It took into account the position of the High Council of Public Health, and the challenges identified by the service thoughtout discussions that occurred between 2013 and 2015 with medical and animal health experts and researchers, health security agencies, institutions, and associations. The draft was then reviewed by the different actors till it reached a point of stable consensus. The plan targets five axes and 15 actions. In what follows, we present how the CK approach can support public health authorities in moving beyond this plan to identify innovative research questions and key actions against LD, as well as to unearth innovative perspectives both for LD specifically and for public health policy design in general. The outcomes of the process were intended for public health authorities as well as potentially researchers, funding agencies, innovators, citizen association planners, and working on this topic.

## Method

### General approach

**Figure 1:**
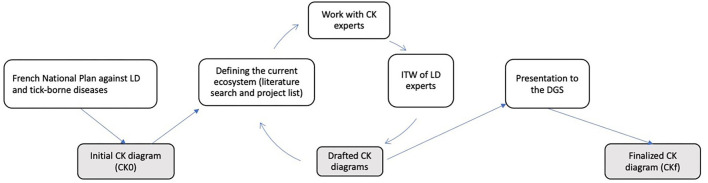
Methodological approach to build the concept-knowledge (CK) diagram to identify innovative research that would be developed to fight Lyme disease in France. LD, Lyme disease; C, concept; K, knowledge; ITW, interviews; DGS, Direction Générale de la Santé (Health General Division of the French Ministry of Health).

We adapted the KCP approach to the context of the Lyme ecosystem, where participants had low availability, were geographically dispersed, and potentially conflicting. We adopted an iterative approach based on knowledge synthesis and exploration with LD experts and CK experts (Figure 1). The whole process was driven by two scientists, one researcher in LD ecology and one researcher in epidemiology and political sciences, both trained in innovative design.

The result of the exploration was illustrated by a CK diagram. A CK diagram is the visual representation of the “knowledge” and “concept” spaces used to explore a concept of interest. The space of “knowledge” is used to grow, organize, and visualize an interdisciplinary base of evidence concerning the topic of study. The space of concepts is used to examine the different design possibilities for the desired object based on factors, such as its characteristics, the actors involved, and how they act. To reflect this step-wise inquiry, concepts are organized in a branching structure. The CK diagram as a whole provides support for the identification of the most promising interventions and for missing pieces of knowledge.

We first created an initial CK diagram (CK0) by analyzing the French Ministry of Health “National Plan Against Lyme Disease” (31). We worked on knowledge associated with the initial concept thanks to the literature search and the analysis of the list of research projects developed on LD ecology, epidemiology, or medicine.

The desirable object we wanted to reach, “An efficient National Plan Against Lyme Disease.” The French national plan had five objectives and 15 “actions”: 1) improve tick surveillance and improve tools to control the tick population. The following actions were identified: risk mapping, assessing the effectiveness of existing tick population management strategies, and assessing the effectiveness of tick repellants. 2) Improve the surveillance and prevention of tick-borne diseases. The following actions were identified: generalized tick surveillance, public information at trailheads, community education, and updating current public information materials. 3) Improve and standardize patient care. The following actions were identified: a coordinated review of tick-borne disease, a national protocol for the diagnosis and plan of care of patients with tick-borne diseases, and the establishment of criteria for the admission of LD as a recognized chronic illness, along with patient rights and a plan of care. 4) Improve diagnostic tests. The following actions were identified: evaluation of existing over-the-counter diagnostic tests and evaluation of the correct interpretation of diagnostic test results. 5) Leverage current research on tick-borne diseases. The following actions were identified: develop new diagnostic tests, better understand the variety of tick-borne pathogens, and establish a prospective patient cohort.

### Literature search

For the literature search, we queried PubMed and Web of Knowledge from 2000 to 2017. In terms of prevention, we searched for tools that target ticks, humans, or animal hosts and classified them according to different principles (chemical, biological, genetic, microbiota, immunologic, environmental, behavioral, and pharmaceutic modifications). Regarding the plan of care, we looked for innovative diagnostic, treatment, and care based the terms “Lyme disease,” “Late Lyme disease,” “Post-Treatment Lyme Disease Syndrome,” and “Chronic Lyme Disease.” We questioned whether the obtained knowledge pushed us to re-organize our entire thought process or open-up concept exploration pathway and looked further for articles accordingly if relevant.

### Research projects

We worked on a list of research projects, run by French research teams, that were active in 2016 and 2017 and that addressed LD, prevention, and ecology. We used the list compiled by the French Group on Ticks and Tick-Borne Diseases, which was created in 2004 to bring scientists from different disciplines to improve knowledge on ticks and tick-borne diseases, as well as the Reference Center for Borrelia and the interviews. It was cross-checked for completeness against a list provided by the French Ministry of Health. Projects were mapped against the CK diagram to identify potential areas that were under-explored.

### Interviews

All the materials (knowledge, project list, identified tools, draft CK diagram) were presented to the LD experts during individual semi-structured interviews. The experts for the interviews were purposefully sampled (32) to maximize the heterogeneity of fields of expertise and reflect the thematic diversity of the challenges posed by LD. We did not receive any refusals. We conducted 10 expert interviews with specialists in *Borrelia* biology and vaccine (*n* = 1), microbiology (*n* = 1), microbiota (*n* = 1), tick genetics and ecology (*n* = 1), forest ecology (*n* = 1), citizen science (*n* = 1), patient involvement (*n* = 1), infectiology (*n* = 1), public health policy (*n* = 2), in addition with two interviews with experts in innovative design. The key questions for the semi-structured interviews were 1) to complete the knowledge regarding possible and existing tools and the list of research projects, and 2) to expand the concept diagram with the new concepts or re-organize the diagram to better reflect or engender the creative process.

### Finalization

We worked back and forth with CK experts and Lyme experts to identify the main concepts and draft a CK diagram. This allowed us to enrich our knowledge base and to work together with CK experts on a more advanced CK diagram. The approach, knowledge, and CK diagram were then presented to the General Health Division of the French Ministry of Health (DGS), which elaborated the national plan against LD. From their feedback and further work with CK experts, we finalized the diagram (CKf). The process was halted after 6 months when the CK diagram was stable through successive steps.

The issue of controllability of design processes is a critical issue, today addressed by advances in design theory (33). The difficulty is in the control of the generative process. Usually, control is focused on the development process or, downstream, on the selection process. It is very difficult to have a “reference” “before” selection/decision that has no bias (or not too strong bias) in the generation process. It was proven that CK theory can provide a reasonable framework to control generativity before selection/decision (34). That is one of the reasons why we relied on design theory as a relevant method to control the generation of alternatives to the question of LD.

We used a qualitative evaluation of V2OR, commonly used in innovative design (35, 36). This method employs a set of four evaluation criteria that are adapted to concept generation: variety, value, originality, and robustness. Variety is obtained by avoiding the proliferation of too many similar ideas. Value refers to the identification of benefits that different actors would get from the innovation. The appearance of surprising properties and the renewal of objects' identity reflect high originality. Robustness refers to the reliability of concept paths, which can be assessed by determining concept resistance to changes in context and by the quality and quantity of knowledge associated with the concept.

## Results

### Input

**Figure 2:**
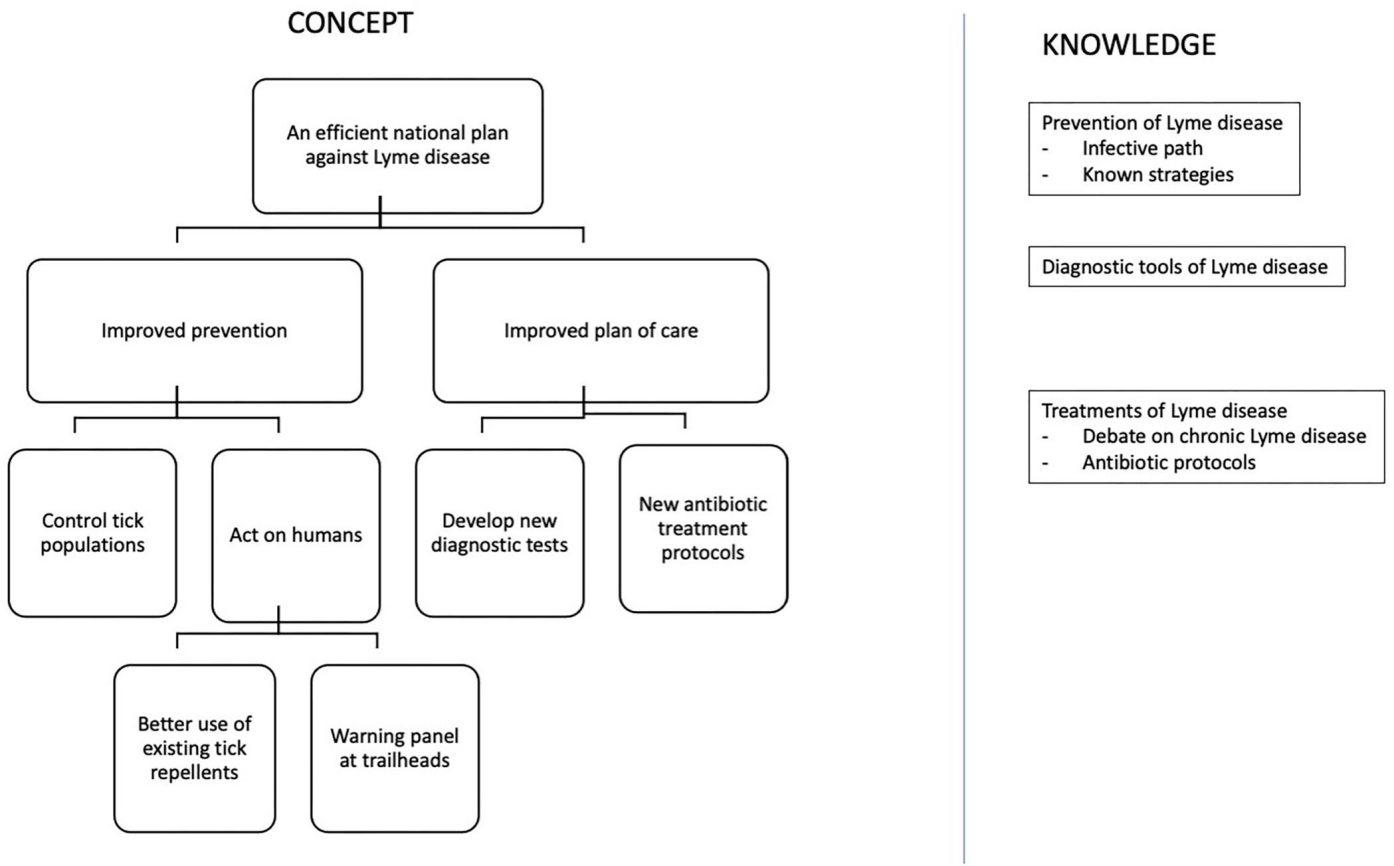
CK diagram of the government plan against Lyme disease (CK0).

Based on the French Ministry of Health's Action Plan against LD, we constructed the initial diagram CK0. In the concept part, improved prevention was then divided into control action on ticks or humans, whereas an improved plan of care presented developed actions on the new diagnostic test and new antibiotic treatments (see Figure 2).

**Figure 3:**
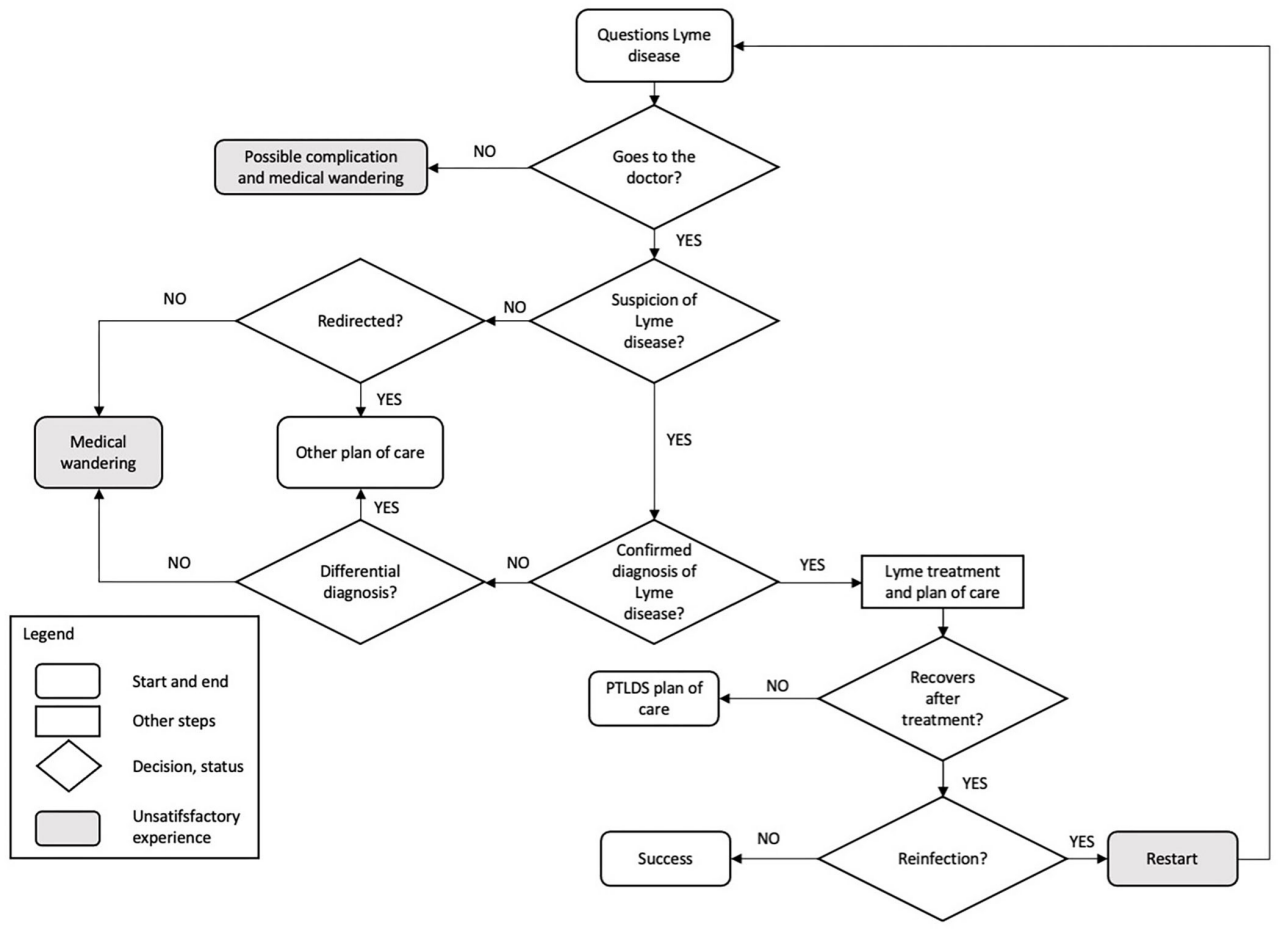
Itinerary of patients who questioned whether they have Lyme disease.

The list of tools for disease prevention, the analysis of the current diagnostic process, and the knowledge around treatment knowledge obtained from the literature search are shown in Supplementary S1. Specifically, prevention tools concerned chemical use or modification of the genetics, microbiota, immunology, environment, neurological and behavioral properties of the target, as well as antibiotic tools. We modeled the itinerary of patients who questions whether they have Lyme in Figure 3. The final list of research projects that were active in 2017, consolidated by the interviews to address LD in France encompassed 18 projects. Most projects were pluriannual projects, with some having started in 2013 or 2014. Seven of them covered ticks and tick bites, three with *Borrelia*, microbiota, and co-infection in ticks, four with concerned knowledge, surveillance, and LD diagnosis, and four with vaccine and care questions (see Supplementary S2). Projects on LD were balanced across prevention, diagnosis, and treatment and across each way of answering challenges within those areas of work.

### Identification of concepts and evolution of the diagram

#### Issues in disease prevention

The prevention of a zoonosis is usually modeled according to its infective path, which includes acaricides to control the tick population, warning panels that inform the population of the risk of tick bite, and vaccination to prevent bacterial infection (CK0).

**Figure 4:**
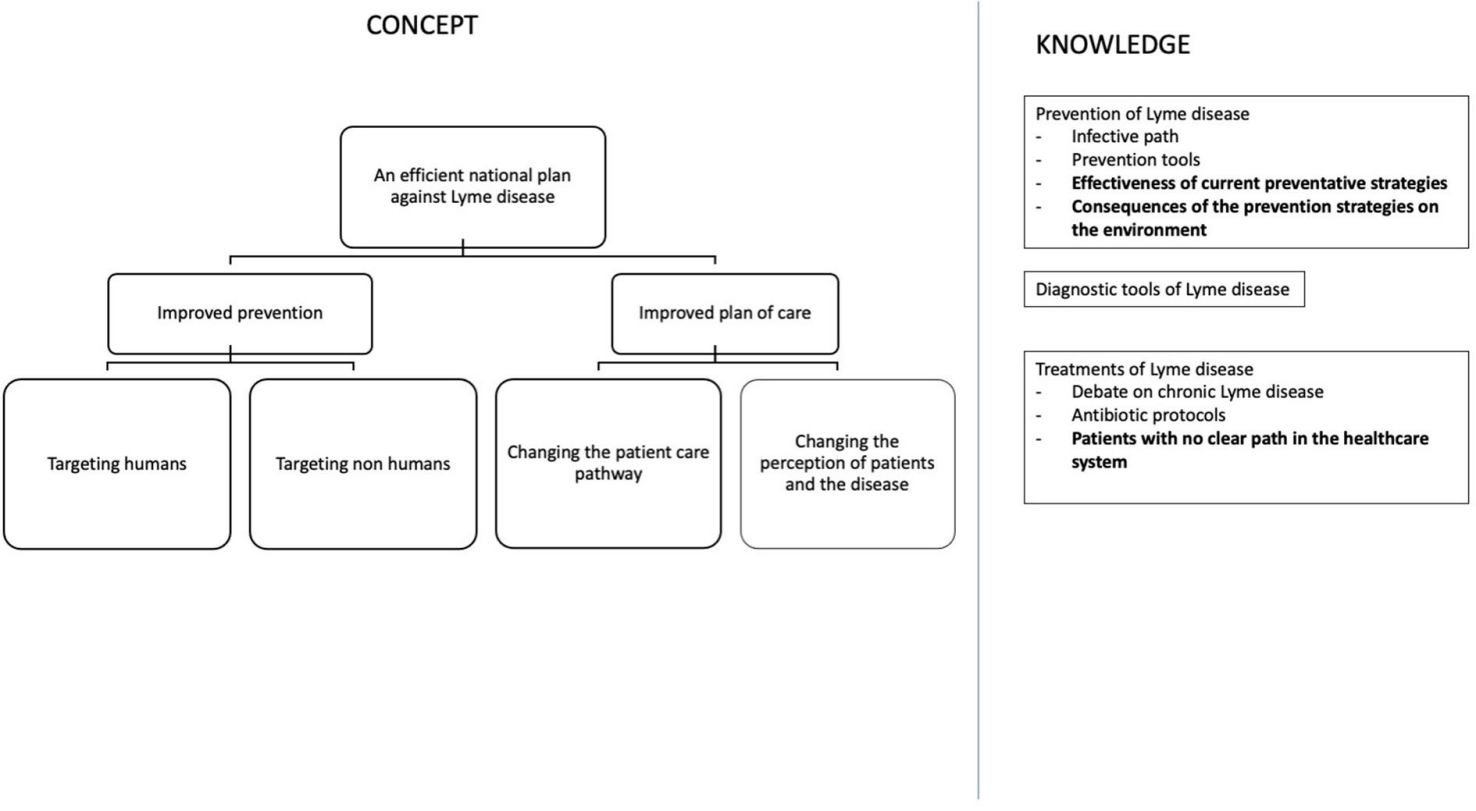
First partitions of the final concept diagram (CKf) and main new mobilized knowledge in bold.

**Figure 5:**
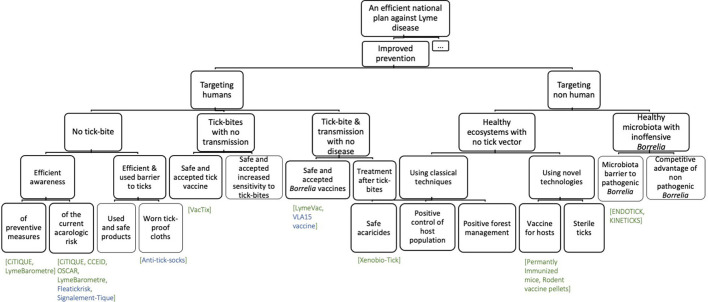
The concept part of the “improved prevention” partition of the CKf. In green, the names of the identified projects listed in Supp. Mat. 2 In blue, the name of networks or products in development.

We found that, in general, tools or actions that target humans were either well-developed but not clearly effective or they were in development with the promise of being effective (Figures 4, 5). The protectiveness of non-acaricidal tick repellants is debated and other actions, like the modification of skin microbiota, require further knowledge. While some tools themselves could be effective, they could prove ineffective in practice because they were used inconsistently, including tucking pants into socks, wearing light-colored clothes, and wearing long-sleeved shirts and long pants (37). Acaricide repulsive (38) and information tools on risk knowledge and tick-proof behavior are two examples of effective tools (39). Furthermore, solutions that target humans include vaccines, which are promising both in terms of effectiveness and their minimal impact on the environment. Vaccines that target ticks have the advantage of blocking pathogen transmission (40). The increased sensitivity to tick bite would help to localize the tick earlier and thus remove it before transmission but requires further knowledge. Regarding preventing the disease while transmitting, vaccines against LD will have to address two challenges: efficacy and vaccine hesitancy, which has been a long-standing challenge, the most recent illustration being the COVID-19 vaccine [e.g., (41–43)]. Systemic treatment after tick bites (one single dose of doxycycline) has proved to be effective against *erythema migrans* occurrence (44). However, French guidelines do not recommend antibiotic prophylaxis for the prevention of LD in endemic areas because the number of patients to be treated in order to avoid one case of *erythema migrans* would be as high as 50 (45).

Finally, studies showed that it was possible to map acarological risk at a landscape or regional scale [e.g., (46)]. These studies prompted the development of projects for apps that provide relevant estimates of the density of questing ticks so that people could be made aware of “high-risk” areas.

Actions that target actors other than humans were either effective and had a measurable but negative impact on the environment, or both their effectiveness and impact on the environment were unclear. The ones that have a known, unwanted impact on the environment are acaricides. Their effectiveness on ticks is demonstrated but difficult to implement over the long term (47–50). Furthermore, they can have sublethal effects on non-target species, such as pollinators or aquatic organisms (51). Landscape management, especially working in forested areas (52–54), and control of deer populations (55) are other techniques but they have to be considered in a whole socio-ecosystem approach (56). The tools that have an unknown impact on LD and/or on the environment include genetically modified hosts and their vaccination (King 2016, Brooks 2016 gray reference in Supplementary S1) and tick pathogens and predators, such as entomopathogenic fungus and wasps [e.g., (57, 58)]. It is unclear that genetically modified rodents would have a significant effect on tick populations, especially if they are released in an open ecosystem with other hosts (59), given that ticks have a much longer lifespan than mosquitoes. The long-term effects of tools acting on other than humans may be politically unacceptable (e.g., culling of deer, deforestation) or very creative and perhaps important to the progress of technology (e.g., permanently immunized mice, GMO ticks), but they are considered impractical due to the difficulty of raising ticks, the important diversity of hosts and bacteria reservoirs and the complexity of the bacteria's ecosystem.

Thus, the CK diagram evolved from CK0 with splitting with action on humans or other than humans and introducing the notion of effectiveness, acceptability, and usage to each path (Figure 4). Regarding the actions on non-humans, we introduced the concept of a healthy ecosystem or microbiota to underline the consideration of the whole system while searching for preventive actions.

#### Issues of the plan of care

**Figure 6:**
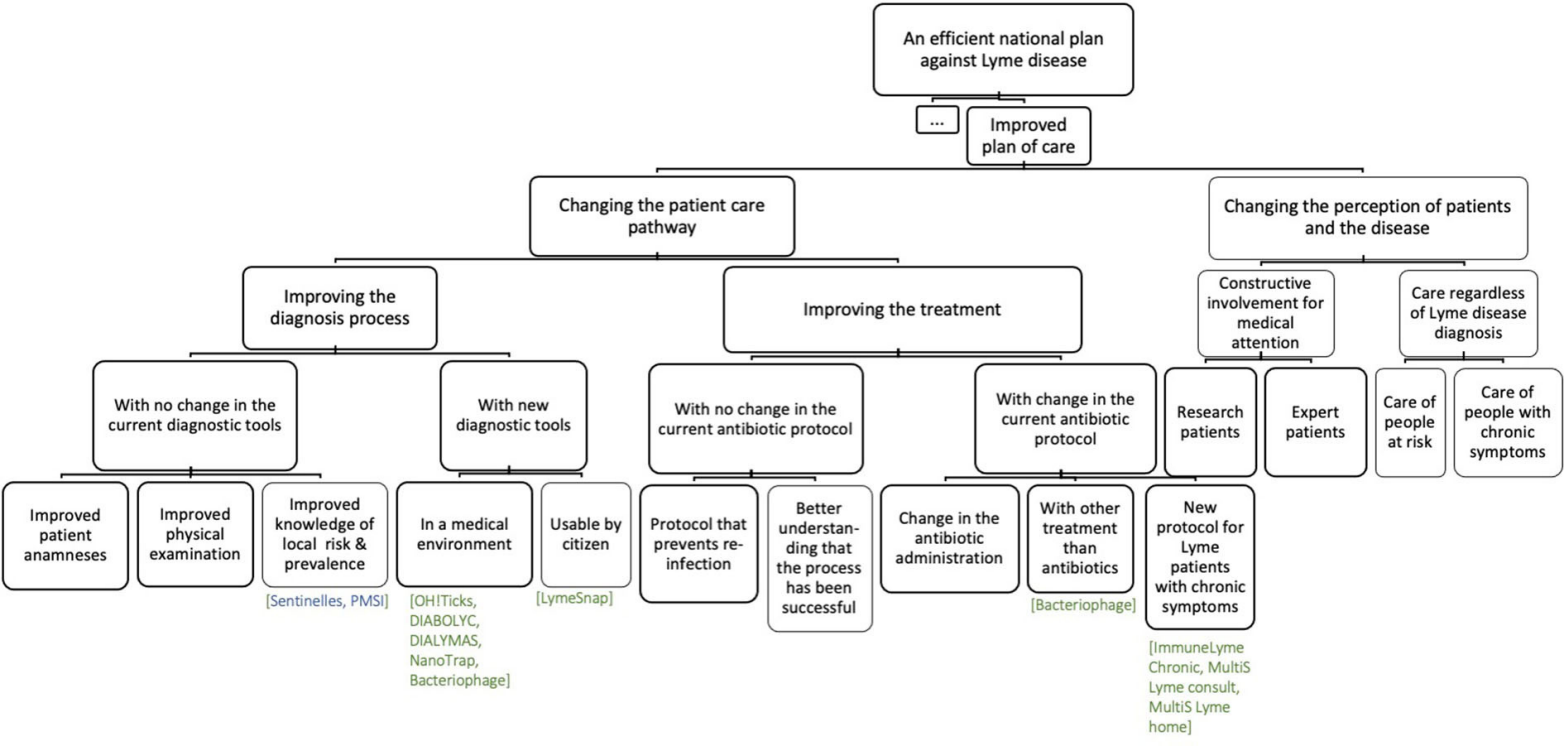
The concept parts of the “improved plan of care” partition of CKf. In green, the names of the identified projects listed in Supp. Mat. 2. In blue, the name of networks or products in development.

The analysis of the literature and the interviews led us to present the patient journey as different steps that can either lead to successful experience or unsatisfactory experience (Figure 3). It qualified symptoms of CLD as an issue that necessitates attention regardless of the evidence, or lack thereof, of the cause of the symptoms (Figure 6).

We organized the concept diagram with a split between changing the patient care pathway and the perception of patients and disease. The improvement of diagnosis with no changes in the current tools calls for better integration of the different sources of information (physical examination, patient history, or local knowledge of risk). Diagnostic tools for LD include serology, intrathecal synthesis of specific anti-Borrelia immunoglobulin, and PCR. The dosage of CXCL13 in the cerebrospinal fluid may be promising but would require subsequent evaluation (60). The use of a prophage-targeting PCR for LB diagnosis is under development (61). In addition to those that are developed for a medical environment, new diagnostic tools could be developed to be usable directly by citizens. Over-the-counter diagnostic test kits have been shown to be inaccurate (62).

Regarding the improvement of treatment, concepts that do not target change in the current antibiotic protocol have been identified along the patient journey. The first concept was to develop protocols to specifically prevent re-infection. To our knowledge, no study has been undertaken that looks into the rates of re-infection in France or the types of people who experience re-infection. Such information could be used to plan projects that mitigate such risks. A second concept would be to improve the patient's understanding of how their own care has been successful. This calls for an improvement in the patient's understanding of their own health status. The other branch regarding the improvement of treatment concerns the testing of new antibiotic protocols or alternative molecules, which include peptides, predatory bacteria, CRISPR, and metals (63), as well as an anti-*Borrelia* phage (64) and characterize their efficacy. It also concerns the development of new protocol for Lyme patients with chronic symptoms.

Concepts regarding the change in the perception of patients and disease addressed the knowledge and the diagnosis status. These concepts were linked to increasing the medical attention to patients with programs that foster their knowledge or involvement in the plan of care, such as co-design plan of care protocol and education to bring the patient to a certain level of expertise. The other concepts concern the care of patients with chronic symptoms independently of Lyme diagnosis. This requires points of entry for multidisciplinary, follow-up, and long-term care.

#### V2OR criteria

The set of concepts that emerged from the analysis demonstrated a high level of variety because they covered the whole CK diagram. Variety also manifested itself in the cross-disciplinary nature of many concepts. For instance, the concepts of effectiveness in preventive actions and health ecosystems require the mobilization of several disciplines. The innovation design process gets most of its value from presenting different ideas based on different disciplines toward a common goal. Thus, it can help different actors build prospective plans of actions. As for originality, few propositions questioned the identity of objects. For instance, the concept of improving the patient's condition regardless of the Lyme diagnosis questions the patient's identity. Indeed, the consideration as the patient does not rely on objective tests, but rather on the overall conditions. The involvement of citizens questions their role as experts or citizen scientists. As well, the importance to consider the impacts of preventive measures on the environment is questioned at what level in a socio-ecosystem a health common goal should be defined. The robustness of the output of the design process is the most difficult to objectify. It is likely that introducing new knowledge (e.g., new health community prevention approach, new vaccine) or shifting ecological conditions (e.g., accentuated climate change, evolution of bacteria or ticks) would modify outcomes. That said, what is proposed in the current CK diagram would remain relevant.

## Discussion

### Four main areas

In the context of ever more complex public health challenges, innovation and multidisciplinary collaboration are desirable but often difficult to achieve. We mobilized CK design theory to identify innovative, cross-sectoral, and cross-disciplinary actions or research questions that address the challenges posed by LD in France. Based on the initial concept of an “efficient plan against LD,” we separated strategies that belong to the realm of prevention and those that belong to the realm of the plan of care, as for the initial national plan of 2016. The analysis of the CK diagram highlighted four main areas: (i) the integration of effectiveness in prevention actions; (ii) the integration of environmental sustainability in prevention actions; (iii) the promotion of constructive involvement of citizens in Lyme challenges; and (iv) the development of care protocols for chronic conditions with an unknown diagnosis.

(i) The integration of effectiveness in actions against LD

Our analysis of the prevention partition showed that many research projects addressed prevention, from action on the environment to vaccines. One step to make them more operational would be to improve the integration of effectiveness in the concepts, i.e., availability, acceptability, use, and efficacy of the proposed concept. This requires understanding how citizens get access to information, integrate it, and modify their behavior (65, 66), such as what has been developed for malaria prevention where for instance, an approach combining mass and interpersonal methods result in the positive improvement of prevention [e.g., (67, 68)]. Septfons et al. (69) reported a trend toward increased knowledge and awareness of tick bites and LD from 2016 to 2019 in France. Indeed, in 2016 66% of participants had heard about LD. They were 79% in 2019. In 2016, 29% of them considered themselves well-informed. The percentage grows up to 41% in 2019. An illustration from our work of a concept that better-highlighted behavior change would be “Installing warning panels at trailheads associated with an effective strategy to make sure that people can follow the recommendations.” This calls for adapting tools to citizen use and understanding the way they perceive their health (70) by associating acarologists with social scientists and ergonomists. Another example would be the development of tick-proof clothes to make sure that they are worn, which suggests possible innovative private–public partnerships. The last example concerned the acarological risk. The information actually depends mainly on research initiatives that model and map acarological risk. From a public policy perspective, France is lacking a national surveillance policy of acarological risk that integrates long-term monitoring, the combination of various sources of information (e.g., citizen data, modeling, active sampling), as well as the needs and expectations of the end-users (stakeholders, citizens, researchers, etc.) (71, 72). The surveillance associated with spatial decision instruments could provide enhanced support for decision-making and management. Such tools have been successfully used for malaria elimination in a variety of countries (73).

With regards to the plan of care, bringing to the patient a better comprehension of their health status would help them to present quicker to the doctor and to better understand the course of the plan of care. Indeed, there is a disruption between the medical and the patient's point of view regarding their health status, especially with chronic symptoms (74). Tools focusing on the knowledge of the patient's own health status are already developed with diseases that require self-care on the part of the patient (e.g., patient decision cards, biofeedback, smartphone apps…) (75, 76). Some tools are being developed to identify *erythema migrans* from pictures based on deep learning approaches (77). They face the challenge of taking into account skin color, picture quality, and finding the right decision criteria for informing end-users of a potential risk (e.g., which likelihood of *erythema migrans* to consider? How to consider the information of risk exposure). They may provide means for patients to report information when consulting doctors (appropriate picture, indication of time and location, etc.) (78). A better knowledge of health status also leads to educating patients in treatment for LD about risk factors and prevention (79).

(ii) The integration of environmental sustainability in prevention actions on LD

The analysis of prevention targeting other living beings called for better integration of ecosystem sustainability from a One Health perspective. The concept is not so much to outline the importance of considering animal reservoirs (80), but rather to move our common goal of prevention from Lyme prevention to a healthy sustainable ecosystem for all living organisms (81). It calls for cross-sectoral and multidisciplinary approaches (82) and leads to research questions regarding the relationship between biodiversity and health at the level of ecosystem or microbiota (83–85). One could imagine designing a “healthy biodiverse territory with no tick-borne transmission” that would be a concrete field laboratory. It could be, for instance, a delimited area, which would concentrate all knowledge and actions on Lyme prevention and plan of care (information, study of efficacy, ecosystem management, diverse prevention tools, etc.). This would require multidisciplinary and multisectoral efforts.

(iii) Promotion of the constructive involvement of citizens in Lyme challenges

Citizens are key in the LD ecosystems. They can be involved in data collection in citizen science programs (e.g., CiTIQUE) to patient organizations that advocate for a different plan of care. Citizen knowledge of LD prevention and care can modify their own behavior but also that of their relatives because people who are sick are very sensitive to advise from relatives. Our work highlights citizen involvement as a tool to move from confrontation to cooperation (86, 87). From data collection to the co-design of prevention or plan of care with expert patients, all these examples of citizen involvement open a new realm of questions that are particularly important in health sciences. We need to further understand the conditions that favor co-design, the learning steps citizen, and how different types of knowledge can be positively combined.

(iv) The development of care protocols for chronic conditions with unknown diagnosis

In 2017, the mindset stated that patients were either positive or negative for LD. Patients who were positive got treatment. Patients who were negative got shuttled into a different part of the healthcare system. This dichotomy breaks down, particularly for people identified at risk, whose infectious status is yet unknown, and people who are clearly unwell with chronic symptoms but do not clearly have LD and do not have a clear path in the healthcare system. As for other chronic conditions for which the physio-pathological pathway is unknown and the plan of care not clearly defined, such as fibromyalgia, chronic fatigue, or long COVID-19, patients with a “diagnosis” of Chronic LD suffer from medical wandering that leads to poor management of the disease, psychological distress, and increased cost of healthcare (88–91). Projects that look at the concept of improvement of patient conditions regardless of the LD diagnosis might draw from previous research in these areas. These projects must be cost-effective for the healthcare system and affordable for the patients (92). The creation of five Tick-Borne Diseases Reference Centers (TBD-RC) in France in 2019 was a step forward in this direction. A multidisciplinary team of experts makes it possible to establish a differential diagnosis and to propose adapted care. Somatoform disorders can benefit from psychological support and adapted rehabilitation. In this context, Raffetin et al. (93) outlined that 70% of the 569 patients who consulted at the center were finally diagnosed with another pathology. Thus, the TBD-RC received a majority of patients who did not have LD.

Developing care protocols for chronic conditions regardless of Lyme diagnosis requires a change in the perception and action of both patients and physicians. The patients need to be able to reconsider the diagnosis (CLD) in which they sometimes strongly believe. Despite the lack of a diagnosis, physicians need to accept and recognize the patient's suffering. It means moving from a disease approach to a syndromic approach, related or not to tick bites. Physicians need improved support to manage this type of syndrome, which is surrounded by unknowns and uncertainties with low scientific evidence. For that purpose, care protocols should be proposed based on multidisciplinary approaches, research protocols, and allopathic and non-allopathic approaches such as rehabilitation.

### Limitations of the study

Our application of CK theory is not an evidence-based and data-driven demonstration of the value of CK theory as a creative tool in the design of public health programs, but rather a pilot application to illustrate the possibilities in a given and limited context.

The distinction between prevention and plan of care stayed stable through successive steps of our study, perhaps pointing to a cultural bias in the literature or for the meta-syntheses and in the sample of interviewees. The CK diagram and design process could be completed by another branch “Simultaneous prevention and plan of care.” Some ideas that may be fostered by considering this point of view have already been highlighted, such as strategies for systemic treatment after tick bite (44) and the strategies to mitigate re-infection. Questioning the dichotomy between prevention and treatment could have value beyond LD as it touches on broad public health and healthcare challenges of improving the affordability, efficacy, and quality in the care of patients with chronic disease or mental health issues. The scope of our study and our access to new expertise limited our ability to look into these concepts in an in-depth manner. Future applications of CK analysis in the public health field might address this possible bias by including dedicated literature and experts in processes.

The number of experts we interviewed was relatively limited but we chose to cross-analyze the output of the expert interviews with the literature and the project analysis. Thanks to our iterative process, we were able to consolidate and complement the information from the different sources and stop the process when the CK diagram was stable with no major new information (94). Nevertheless, exploring more deeply the proposed solutions can require the mobilizing of new experts who can expand the available knowledge.

We reached what we could call a referential of concepts that address LD issues. We did not develop to the project phase, where concepts are selected for their potential interest and the means needed to address the selected concepts are identified, i.e., which actors should be involved, what steps should be planned, what resources are needed, what research would be relevant, etc. At this stage, the feasibility of each path could not be proven, i.e., solutions that might call for additional work. The same is valid for the translational potential: the “market” or “user value” could not be proven, and at the same time it was also impossible to prove that there was no value.

Altogether, the type of work we conducted could contribute to the cost/benefit analysis of public health and medical measures in several ways. First, for each innovation path, it is possible to have a specific cost/benefit analysis adapted to the specific proposed innovation. Second, this type of analysis can underline new possible benefit criteria or cost criteria that should be taken into account in future. For instance, this work highlighted the potential benefit of a database of well-recorded images of *erythema migrans* and their evolution over time. Third, the cost/benefit analysis of the study itself could be estimated: the cost is non-negligible (e.g., several of man.month) and the benefit has to be estimated in terms of (i) a clearer picture of the innovation field (variety of approaches, of expertise,…) that encompasses more directions than the National Plan against LD, (ii) capacity to monitor the variety of projects, led by a variety of institutional actors (research institutes, companies, practitioners…), and (iii) identification of paths that are orphan of any exploration and design efforts and yet might be relevant for the society. Although difficult to quantify, the overall benefit seems high and obtained at a relatively limited cost.

### Perspective-conclusion

Two reports elaborated by groups of French parliamentarians were published in 2021. They addressed the funding and efficacy of, for one the action plan against LD and for the other the plan of care for patients (95, 96). They pointed out the necessity to continue the actions of the 2016 plan with regards to information campaigns, awareness of acarological risk, and tick surveillance; the need to structure the patient journey from the generalist to the TBD-RC; and the need to increase fundamental knowledge on tick-borne and chronic symptoms associated with Lyme. Both reports highlighted the necessity for increased funding and inter-sectoral action. Our work goes beyond these recommendations toward a broader perspective together with a better understanding of the link between the different concepts. From a design point of view, by working on topics while freeing ourselves from fixation and known contention, we changed the identity of some objects, such as the patient identity, the citizen involvement, and health common goal (from population health to ecosystem health). This means integrating ecological sciences and humanities from the start as well as valuing a multidisciplinary approach to patients' medical journey. The process could be further pursued to help guide a group learning process that made intentional space for expert and local knowledge (15). CK theory could be a promising frame to assist public health professionals design programs in complex, yet urgent contexts where research and data collection are not yet sufficient to provide clear guidance.

## Data availability statement

The raw data supporting the conclusions of this article will be made available by the authors, without undue reservation.

## Ethics statement

Ethical review and approval was not required for the study on human participants in accordance with the local legislation and institutional requirements. The patients/participants provided their written informed consent to participate in this study.

## Author contributions

GV, PL, BW, and OL designed the study. KU did the literature search, the interviews and the work on the design diagrams. GV, KU, EB, PF-K, PL, BW, and OL worked on the analyzes and interpretation of the study. GV and KU drafted the manuscript. All authors reviewed the manuscript.

## Funding

The research position of KU was founded by the Chair of Design Theory and Methods for Innovation.

## Conflict of interest

The authors declare that the research was conducted in the absence of any commercial or financial relationships that could be construed as a potential conflict of interest.

## Publisher's note

All claims expressed in this article are solely those of the authors and do not necessarily represent those of their affiliated organizations, or those of the publisher, the editors and the reviewers. Any product that may be evaluated in this article, or claim that may be made by its manufacturer, is not guaranteed or endorsed by the publisher.
