# Supplementary material for: Design theory to better target public health priorities: An application to Lyme disease in France
Source: Front Public Health. 2022 Nov 7;10:980086. doi: 10.3389/fpubh.2022.980086 (PMC9676681; doi:10.3389/fpubh.2022.980086)
Supplement: Supplementary file 1 [file Data_Sheet_1.docx]

# Supplementary Material of the CK Lyme paper

## Suppl. Mat. 1: Knowledge from literature search on existing tools to prevent Lyme disease, diagnosis and treatment (2017)

### Tools for prevention by action on the ticks, the bacteria, the tick and reservoirs and the humans

The most common efforts to control tick populations employ acaricides that are either applied sprayed in the environment or applied to tick hosts. The effectiveness of acaricides applied to the environment is documented (1), while the effectiveness of acaricides on tick hosts is debated (1-3). Landscape and vegetation management are another technique (4-6) along with control of deer populations (7, 8), but need to be in place with a socio-ecosystem perspective. To our knowledge there is no known resistance of *I. ricinus* to acaricides. Resistance of other ticks to acaricides is well documented (9).

Other means include genetically modified hosts (King 2016, grey reference), genetically modified ticks (Brooks 2016 grey reference) and entomopathogenic fungus and nematodes studied on *Ixodes* or other tick genus (10-13).

Other than tick control, efforts to prevent Lyme disease come from preventing tick bites and *Borrelia* transmission. Public health officials often recommend the public tuck pants into socks, wear light colored clothing and use insect repellent when going in to the tick-infected forests. These measures have either been shown to make no difference or they have yielded different results in different studies (14). Prophylactic antibiotic treatment after tick bites has been shown to reduce the risk to develop Lyme borreliosis (15, 16). In 1998, GalaxoSmithKline released a vaccine against Lyme disease. The vaccine was withdrawn from the market in 2002. Arthritic and neuropathological auto-immune reactions potentially associated with the vaccine were reported. The vaccine might have targeted antigens that are thought to cause autoimmune reactions (14). Valneva in 2017 will move a new potential vaccine to phase 1 trials.

**Table S1:** List of the possible tools to prevent Lyme disease.

| **Target** | **Examples** | **Feasibility** | **Effectiveness** | **Limits** | **Ref.** |
| --- | --- | --- | --- | --- | --- |
| Chemical use | |  |  |  |  |
| Ticks | Acaricides in the environment | Tested | Localized, short-term | Toxicity in the environmental | (1) |
| Hosts | Pour-on or baits acaricides on wild ungulates or rodents | Tested | Demonstrated efficiency locally | Possible evolution of resistance; unclear impact on the environment and other species | (2, 3, 17) |
| Humans | Acaricide repulsive | Already exist | Demonstrated efficiency | Possible evolution of resistance | (18) |
| Biological control | |  |  |  |  |
| Ticks | Entomopathogen fungus | Lab experimentation, in delimited field area | Efficiency demonstrated in experiments; poor and unstable efficiency in field | Long-term consequences not known | (11, 19) |
|  | Parasitoids (*Ixodiphagus hookeri*) | Experimental and theoretical studies | Debated efficiency; low effect on population size | Unknown consequences of massive introduction of parasitoids | (20, 21) |
|  | Vertebrate predators | In delimited area | Efficiency shown under some conditions | Effect on the ecosystem have to be measured if introduced or favored | (22) |
|  | Parasites (nematods) | Laboratory experiment | Observed mortality | Unknown ecosystem effect if introduced | (11) |
| Genetic modifications | |  |  |  |  |
| Bacteria | Introduce GMO bacteria with low pathogenicity | To be tested | Existing knowledge of the infection mechanisms; Need selection advantage of the GMO | Unknown consequences in the ecosystem |  |
| Ticks | GMO ticks (including sterilized males and transgenic ticks unable to transmit *Borrelia*) | Lab experiment on other genus | Very difficult to apply in the field (low dispersion of ticks, breed difficulties, etc.) | *id* | Brooks 2016* |
| Hosts | Permanently immunized mice | Experimental studies | To be determined | *id* | King 2016** |
| Microbiome modifications | |  |  |  |  |
| Ticks | Modification of microbiota | To be tested | Difficulty to modify a substantial part of the population | To be determined | (23) |
| Humans | Modification of skin microbiota to repel ticks | Explored for mosquitoes | To be determined | To be determined | (24) |
| Immunologic modifications | |  |  |  |  |
| Hosts | Edible *Borrelia* vaccine in reservoir hosts | Tested for rodents in the field | Reduction of nymphal infestation in the environment (23-76%) | Need to target major reservoir hosts (not so evident in Europe); Maintenance of collective immunity over the long-term | (25, 26) |
|  | Tick vaccine in reservoirs hosts | Experimental testing | Reduction of tick infestation (53%) | Implementation in the field | (27) |
| Humans | *Borrelia* vaccine | Not approved | Low efficiency because of antigenic modification during the infection | Symptomatic secondary effects | (28-30) |
|  | Tick vaccine against salivary antigens or other antigens | Experimental testing | Low effectivity of antigens | Protective efficiency | (31) |
| Environmental modification | |  |  |  |  |
| Ticks | Vegetation and landscape management | Field experiments | Shrub clearing and control of Japanese barberry reduced the number of ticks infected, trail management | Modification of biodiversity | (4-6) |
| Hosts | Deer exclusion | Case studies | Demonstrated; | Difficulties to maintain on the long term | (7, 8) |
|  | Deer hunting | Management measures | Efficiency of deer population control in different context | Hunting feasibility and acceptability | (32) |
|  | Reservoir mouse control | Observational studies | Density reduction of major reservoir host correlates with lower risk | Ethics for reservoir host control | (33) |
|  | Favor host diversity | Observational studies and modeling | Low forest fragmentation and predator presence reduce infected ticks | Modification of complex host communities in natural areas | (34, 35) |
| Neurological and behavioral modifications | |  |  |  |  |
| Ticks | Modify or lure sensorial system of the ticks | To be determined | To be determined | To be determined | (36) |
| Humans | Favor risk knowledge and tick-proof behavior | Recommendation, boards | Poor observance or recommendation, heterogeneous knowledge | Access and appropriation of information | (14, 37) |
| Antibiotic modifications | |  |  |  |  |
| Reservoirs | Doxycycline baits for rodents | Experimental trials | Reduction in host infection rate | Evolution of resistance; long-term consequences | (38) |
| Humans | Preventive antibiotic treatment after tick-bites | Experimental trials | Risk reduction of developing Lyme disease | Evolution of resistance | (15, 16) |

* Brooks, D. 2016. Could we genetically modify ticks so they don’t pass on Lyme disease? Maybe, but …. [Online retrieved from <https://granitegeek.concordmonitor.com/2016/02/09/could-we-genetically-modify-ticks-so-they-dont-pass-on-lyme-disease-maybe-but/> ].

** King, B. J. 2016. Are genetically engineered mice the answer to combating Lyme disease? [Online retrieved from <https://www.npr.org/sections/13.7/2016/06/16/482279851/are-genetically-engineered-mice-the-answer-to-combating-lyme-disease> ].

### Tools for Lyme disease diagnosis

The most common laboratory diagnostic test for Lyme disease is a two-step serology. In France, it is common to use an ELISA as the first-line test and then to follow-up positive or uncertain results with a Western Blot. This two-step serology is cost-saving compared to using just the Western blot. The ELISA and the Western blot have comparable sensitivities. The ELISA is cheaper, making it a better screening test. The Western Blot is more specific, making it indispensable for confirmation of diagnosis (39).

PCR technology on blood and serum samples are not useful, as Lyme *Borrelia* is rapidly cleared from blood. Some laboratories offer culture methods to diagnose Lyme disease, but the CDC has strongly cautioned against the use of such cultures due to the high number of false-positives from poor laboratory technique and misinterpretation of results (40, 41).

All studies show the lack of sensibility of the two-step diagnosis at the early stage of Lyme disease. Serological tests are not recommended at this stage and diagnosis needs to rely on the presence of EM. Medical practitioners are advised to monitor patients with a recent history of tick-bites and/or recent symptoms associated with Lyme disease but without evidence of EM. Over-the-counter diagnostic test kits have been shown to be inaccurate (42). In a study in the Appalachian trail, Knoll et al have shown that hikers are poorly able to recognize *erythema migrans* on photographies, but are highly exposed (37).

Efforts to improve the sensitivity of diagnosis tools in early stages of Lyme disease are in development but not yet validated, such as the dissociation of complexed antibodies in early Lyme disease (43), two-tier ELISA (44, 45), and immune-PCR test (iPCR) (46, 47).

### Tools for Lyme disease impact and symptoms

#### Public health impact

In France, the annual incidence of Lyme disease was estimated at over 50,00 people/year in 2016 (48). The Réseau Sentinelles - a government-run, cooperation between general practitioners and public health officials - has been collecting, compiling and analyzing data on Lyme disease since 2009. The disease is not notifiable disease in France. The economic impact of Lyme disease has not been estimated in France. It was estimated in the United States at 203 million dollars per year in the United States (49). A national action plan to improve the prevention, diagnosis and treatment of Lyme disease in France was launched in 2016 (50).

#### Clinical presentation & progression

Lyme disease is caused by the pathogenic tick-borne spirochete of *Borrelia burgdorferi* group (“Lyme *Borrelia*”): *B. burgdorferi* sensu stricto, *B. afzelii*, *B. bavariensis, B. garinii*, *B. valesiana* and *B. spielmanii*. The most common clinical indicator of infection by Lyme *Borrelia* is *erythema migrans* (EM) (51). EM in layman’s terms is a bull’s eye rash, where a first lesion grows in a ring-like fashion to form a rash which resembles a target. The EM is the first symptom of Lyme disease. It is not always seen, or recognized. The rash is indicative that the bacteria is progressing at the cutaneous level. In a later stage of the disease, the bacteria migrate throughout the body. It invades synovial and cerebrospinal fluids preferentially; causing what is designated as Lyme arthritis and Neuroborreliosis respectively. In a minority of cases, the bacteria can infect the heart and the skin causing carditis (inflammation of the heart) and what is known as acrodermatitis chronica atrophicans (ACA). Berglund et al. (52) conducted an epidemiological study of Lyme disease in Southern Sweden. The disease was made notifiable for a year during 1992 to 1993 in the region. Investigators retained patients that presented an EM or that presented one more clinical signs and a positive serologic test. 1,471 patients (0,69% of inhabitants) fulfilled these requirements. Of the confirmed cases, 79% of the patients remembered a tick bite, 77% presented an EM, 16% had neuroborreliosis, 7% had arthritis, 3% had ACA and less than 1% had carditis. None of these patients had previously been treated with antibiotics.

### Tools for Lyme disease treatment

Lyme disease is treated with antibiotic therapy, usually doxycycline. The recommended treatment is for 2 to 4 weeks depending on the clinical presentation (53-55).

Many alternative antibiotic treatments have not been successful, such as long-term antibiotic therapies (56). The effectiveness of hydroxychloroquine, tinindazole and telithromycin on Lyme *Borrelia* have been investigated in vitro though the relevance of these studies to Lyme disease in humans have been debated (57-60). Plasmapherisis was successful in clinical case reports (61) and yet caused the death of a patient in another case report (62). Anti-*Borrelia* phage have been identified (63) but only used experimentally. Alternative groups of patients and medical professionals, some of them organized in associations, provide not specific recommendations on the nature of the molecules and the duration of treatment, which are supposed to be based on the physician’s experience and the patient clinical response (64, 65), which has been denounced (66, 67). Thus, in some conditions they advocate for the legalization and widespread use of longer alternative antibiotic therapies. Meta-analysis studies find no significant differences between antibiotic therapies ranging from 4 to 12 weeks and the established treatment protocols (53, 54).

***Reference cited***

1. Jordan RA, Schulze TL, Eisen L, Dolan MC. Ability of Three General-Use Pesticides to Suppress Nymphal *Ixodes Scapularis* and *Amblyomma Americanum* (Acari: Ixodidae). *Journal of the American Mosquito Control Association* (2012) 33(1):50-5. doi: <http://dx.doi.org/10.2987/16-6610.1>.

2. Carroll JF, Allen PC, Hill DE, Pound JM, Miller JA, George JE. Control of *Ixodes Scapularis* and *Amblyomma Americanum* through Use of the '4-Poster' Treatment Device on Deer in Maryland. *Exp Appl Acarol* (2002) 28(1-4):289-96.

3. Carroll JF, Pound JM, Miller JA, Kramer M. Sustained Control of Gibson Island, Maryland, Populations of *Ixodes Scapularis* and *Amblyomma Americanum* (Acari: Ixodidae) by Community-Administered 4-Poster Deer Self-Treatment Bait Stations. *Vector Borne Zoonotic Dis* (2009) 9(4):417-21. doi: 10.1089/vbz.2008.0166.

4. Williams SC, Ward JS. Effects of Japanese Barberry (Ranunculales: Berberidaceae) Removal and Resulting Microclimatic Changes on *Ixodes Scapularis* (Acari: Ixodidae) Abundances in Connecticut, USA. *Environmental Entomology* (2010) 39:1911–21.

5. Tack W, Madder M, Baeten L, Vanhellemont M, Verheyen K. Shrub Clearing Adversely Affects the Abundance of *Ixodes Ricinus* Ticks. *Exp Appl Acarol* (2013) 60(3):411-20. Epub 2013/01/25. doi: 10.1007/s10493-013-9655-0.

6. Verheyen K, Ruyts SC. *How Can Forest Managers Help to Reduce the Risk for Lyme Borreliosis?* M A H Braks SEvW, W Takken, H Sprong, editor(2016). 233-41 p.

7. Daniels TJ, Fish D, Schwartz I. Reduced Abundance of *Ixodes Scapularis* (Acari: Ixodidae) and Lyme Disease Risk by Deer Exclusion. *Journal of Medical Entomology* (1993) 30(6):1043-9. doi: <https://doi.org/10.1093/jmedent/30.6.1043>.

8. Rand PW, Lubelczyk C, Holman MS, Lacombe EH, Smith RP. Abundance of *Ixodes Scapularis* (Acari: Ixodidae) after the Complete Removal of Deer from an Isolated Offshore Island, Endemic for Lyme Disease. *Journal of Medical Entomology* (2004) 41(4):779-84.

9. Li AY, Davey RB, Miller RJ, George JE. Detection and Characterization of Amitraz Resistance in the Southern Cattle Tick, *Boophilus Microplus* (Acari: Ixodidae). *J Med Entomol* (2004) 41:193-200.

10. Kaaya GP, Mwangi EN, Ouna EA. Prospects for Biological Control of Livestock Ticks, *Rhipicephalus Appendiculatus* and *Amblyomma Variegatum*, Using the Entomogenous Fungi *Beauveria Bassiana* and *Metarhizium Anisopliae*. *Journal of Invertebrate Pathology* (1996) 67(1):15-20. doi: <https://doi.org/10.1006/jipa.1996.0003>.

11. Hartelt k, Wurst E, Collatz J, Zimmermann G, Kleespies RG, Oehme RM, et al. Biological Control of the Tick *Ixodes Ricinus* with Entomopathogenic Fungi and Nematodes: Preliminary Results from Laboratory Experiments. *International Journal of Medical Microbiology* (2008) 298(1):314-20. doi: <https://doi.org/10.1016/j.ijmm.2007.10.003>.

12. Samish M, Rehacek J. Pathogens and Predators of Ticks and Their Potential in Biological Control. *Annual Review of Entomology* (1999) 44:159-82. doi: <https://doi.org/10.1146/annurev.ento.44.1.159>.

13. Samish M, Ginsberg H, Glazer I. Biological Control of Ticks. *Parasitology* (2005) 129(S1):S389-S403. Epub 04/19. doi: 10.1017/S0031182004005219.

14. Institute-of-Medicine. *Critical Needs and Gaps in Understanding Prevention, Amelioration, and Resolution of Lyme and Other Tick-Borne Diseases: The Short-Term and Long-Term Outcomes: Workshop Report*. Washington (DC): National Academies Press (2011).

15. Warshafsky S, Lee DH, Francois LK, Nowakowski J, Nadelman RB, Wormser GP. Efficacy of Antibiotic Prophylaxis for the Prevention of Lyme Disease: An Updated Systematic Review and Meta-Analysis. *J Antimicrob Chemother* (2010) 65(6):1137-44. Epub 2010/04/13. doi: 10.1093/jac/dkq097.

16. Schwameis M, Kündig T, Huber G, von Bidder L, Meinel L, Weisser R, et al. Yopical Azithromycin for the Prevention of Lyme Borreliosis: A Randomised, Placebo-Controlled, Phase 3 Efficacy Trial. *The Lancet Infectious Disease* (2016) 17(3):322-9. doi: <https://doi.org/10.1016/S1473-3099(16)30529-1>.

17. Dolan MC, Maupin GO, Schneider BS, Denatale C, Hamon N, Cole C, et al. Control of Immature *Ixodes Scapularis* (Acari: Ixodidae) on Rodent Reservoirs of *Borrelia Burgdorferi* in a Residential Community of Southeastern Connecticut. *Journal of Medical Entomology* (2004) 41(6):1043-54.

18. Pages F, Dautel H, Duvallet G, Kahl O, de Gentile L, Boulanger N. Tick Repellents for Human Use: Prevention of Tick Bites and Tick-Borne Diseases. *Vector-Borne and Zoonotic diseases* (2014) 14(2):85-93. doi: 10.1089/vbz.2013.1410.

19. Stafford KC, 3rd, Allan SA. Field Applications of Entomopathogenic Fungi *Beauveria Bassiana* and *Metarhizium Anisopliae* F52 (Hypocreales: Clavicipitaceae) for the Control of *Ixodes Scapularis* (Acari: Ixodidae). *Journal of Medical Entomology* (2010) 47(6):1107-15.

20. Knipling EF, Steelman CD. Feasibility of Controlling *Ixodes Scapularis* Ticks (Acari : Ixodidae), the Vector of Lyme Disease, by Parasitoid Augmentation. *Journal of Medical Entomology* (2000) 37(5):645-52. doi: 10.1603/0022-2585-37.5.645.

21. Collatz J, Selzer P, Fuhrmann A, Oehme RM, Mackenstedt U, Kahl O, et al. A Hidden Beneficial: Biology of the Tick-Wasp Ixodiphagus Hookeri in Germany. *Journal of Applied Entomology* (2011) 135(5):351-8. doi: 10.1111/j.1439-0418.2010.01560.x.

22. Keesing F, Brunner J, Duerr S, Killilea M, LoGiudice K, Schmidt K, et al. Hosts as Ecological Traps for the Vector of Lyme Disease. *Proceedings of the Royal Society B-Biological Sciences* (2009) 276(1675):3911-9. doi: 10.1098/rspb.2009.1159.

23. Narasimhan S, Fikrig E. Tick Microbiome: The Force Within. *Trends Parasitol* (2015) 31(7):315-23. Epub 2015/05/06. doi: 10.1016/j.pt.2015.03.010.

24. Verhulst NO, Takken W, Dicke M, Schraa G, Smallegange RC. Chemical Ecology of Interactions between Human Skin Microbiota and Mosquitoes. *FEMS Microbiol Ecol* (2010) 74(1):1-9. Epub 2010/09/16. doi: 10.1111/j.1574-6941.2010.00908.x.

25. Richer LM, Brisson D, Melo R, Ostfeld RS, Zeidner N, Gomes-Solecki M. Reservoir Targeted Vaccine against *Borrelia Burgdorferi*: A New Strategy to Prevent Lyme Disease Transmission. *J Infect Dis* (2014) 209(12):1972-80. Epub 2014/02/14. doi: 10.1093/infdis/jiu005.

26. Gomes-Solecki M. Blocking Pathogen Transmission at the Source: Reservoir Targeted Ospa-Based Vaccines against *Borrelia Burgdorferi*. *Front Cell Infect Microbiol* (2014) 4:136. Epub 2014/10/14. doi: 10.3389/fcimb.2014.00136.

27. Bensaci M, Bhattacharya D, Clark R, Hu LT. Oral Vaccination with Vaccinia Virus Expressing the Tick Antigen Subolesin Inhibits Tick Feeding and Transmission of *Borrelia Burgdorferi*. *Vaccine* (2012) 30(42):6040-6. Epub 2012/08/07. doi: 10.1016/j.vaccine.2012.07.053.

28. Kaaijk P, Luytjes W. Vaccination against Lyme Disease: Are We Ready for It? *Hum Vaccin Immunother* (2016) 12(3):757-62. doi: 10.1080/21645515.2015.1087628.

29. Nardelli D, Munson E, Callister SM, Schell RF. Human Lyme Disease Vaccines: Past and Future Concerns. *Future Microbiology* (2009) 4(4):457-69. doi: 10.2217/fmb.09.17.

30. Plotkin SA. Need for a New Lyme Disease Vaccine. *N Engl J Med* (2016) 375(10):911-3. doi: 10.1056/NEJMp1607146.

31. Merino O, Alberdi P, Perez de la Lastra JM, de la Fuente J. Tick Vaccines and the Control of Tick-Borne Pathogens. *Front Cell Infect Microbiol* (2013) 3:30. Epub 2013/07/13. doi: 10.3389/fcimb.2013.00030.

32. Gilbert L, Maffey GL, Ramsay SL. The Effect of Deer Management on the Abundance of Ixodes Ricinus in Scotland. *Ecological Applications* (2012) 22(2).

33. Sinski E, Pawelczyk A, Bajer A, Behnke JM. Abundance of Wild Rodents, Ticks and Environmental Risk of Lyme Borreliosis: A Longitudinal Study in an Area of Mazury Lakes District of Poland. *Annals of Agricultural and Environmental Medicine* (2006) 13(2):295-300.

34. Hofmeester TR, Jansen PA, Wijnen HJ, Coipan EC, Fonville M, Prins HHT, et al. Cascading Effects of Predator Activity on Tick-Borne Disease Risk. *Proceedings of the Royal Society B: Biological Sciences* (2017) 284(1859):20170453. doi: 10.1098/rspb.2017.0453.

35. Allan BF, Keesing F, Ostfeld RS. Effect of Forest Fragmentation on Lyme Disease Risk. *Conserv Biol* (2003) 17(1):267-72.

36. Carr AL, Mitchell RD, III, Dhammi A, Bissinger BW, Sonenshine DE, Roe RM. Tick Haller's Organ, a New Paradigm for Arthropod Olfaction: How Ticks Differ from Insects. *Int J Mol Sci* (2017) 18(7). Epub 2017/07/19. doi: 10.3390/ijms18071563.

37. Knoll JM, Ridgeway AC, Boogaerts CM, Burket GA, III. Appalachian Trail Hikers’ability to Recognize Lyme Disease by Visual Stimulus Photographs. *Wilderness & Environmental Medicine* (2014) 25(1):24-8. doi: 10.1016/j.wem.2013.09.009.

38. Dolan MC, Schulze TL, Jordan RA, Schulze CJ, Ullmann AJ, Hojgaard A, et al. Evaluation of Doxycycline-Laden Oral Bait and Topical Fipronil Delivered in a Single Bait Box to Control Ixodes Scapularis (Acari: Ixodidae) and Reduce Borrelia Burgdorferi and Anaplasma Phagocytophilum Infection in Small Mammal Reservoirs and Host-Seeking Ticks. *J Med Entomol* (2017) 54(2):403-10. Epub 2016/12/25. doi: 10.1093/jme/tjw194.

39. Mavin S, McDonagh S, Evans R, Milner RM, Chatterton JMW, Ho-Yen DO. Interpretation Criteria in Western Blot Diagnosis of Lyme Borreliosis. *British Journal of Biomedical Science* (2011) 68(1):5-10. doi: 10.1080/09674845.2011.11732834.

40. Eshoo MW, Crowder CC, Rebman AW, Rounds MA, Matthews HE, Picuri JM, et al. Direct Molecular Detection and Genotyping of *Borrelia Burgdorferi* from Whole Blood of Patients with Early Lyme Disease. *PLoS One* (2012) 7(5):e36825. doi: 10.1371/journal.pone.0036825.

41. Nelson C, Hojvat S, Johnson B, Petersen J, Schriefer M, Ben Beard C, et al. Concerns Regarding a New Culture Method for *Borrelia Burgdorferi* Not Approved for the Diagnosis of Lyme Disease. *Morbidity and Mortality Weekly Report* (2014) 65(15):333-.

42. Cook MJ, Puri BK. Commercial Test Kits for Detection of Lyme Borreliosis: A Meta-Analysis of Test Accuracy. *International Journal of General Medicine* (2016) 9:427-40. doi: 10.2147/IJGM.S122313.

43. Brunner M, Sigal LH. Immune Complexes from Serum of Patients with Lyme Disease Contain *Borrelia Burgdorferi* Antigen and Antigen-Specific Antibodies: Potential Use for Improved Testing. *J Infect Dis* (2000) 182(2):534-9. doi: 10.1086/315724.

44. Branda JA, Linskey K, Kim YA, Steere AC, Ferraro MJ. Two-Tiered Antibody Testing for Lyme Disease with Use of 2 Enzyme Immunoassays, a Whole-Cell Sonicate Enzyme Immunoassay Followed by a Vlse C6 Peptide Enzyme Immunoassay. *Clin Infect Dis* (2011) 53(6):541-7. doi: 10.1093/cid/cir464.

45. Wormser GP, Levin A, Soman S, Adenikinju O, Longo MV, Branda JA. Comparative Cost-Effectiveness of Two-Tiered Testing Strategies for Serodiagnosis of Lyme Disease with Noncutaneous Manifestations. *J Clin Microbiol* (2013) 51(12):4045-9. doi: 10.1128/JCM.01853-13.

46. Halpern MD, Jain S, Jewett MW. Enhanced Detection of Host Response Antibodies to *Borrelia Burgdorferi* Using Immuno-Pcr. *Clin Vaccine Immunol* (2013) 20:350-7.

47. Theel ES. The Past, Present, and (Possible) Future of Serologic Testing for Lyme Disease. *J Clin Microbiol* (2016) 54(5):1191-6. doi: 10.1128/JCM.03394-15.

48. Sentinelles. Bilan Annuel Du Réseau Sentinelles. Institut Pierre Louis d’Epidémiologie et de Santé Publique, UMR S 1136, Institut National de la Santé et de la Recherche Médicale (INSERM), Université Pierre et Marie Curie (UPMC), (2016).

49. Zhang X, Meltzer MI, Peña CA, Hopkins AB, Wroth L, Fix AD. Economic Impact of Lyme Disease. *Emerging Infectious Diseases* (2006) 12(4):653-60.

50. MASS. Plan National De Prévention Et De Lutte Contre La Maladie De Lyme Et Les Maladies Transmissibles Par Les Tiques. Ministère des Affaires Sociales et de la Santé - République Française, (2016) Septembre 2016. Report No.

51. CDC. Lyme Disease (*Borrelia Burgdorferi*) 2017 Case Definition (2017). Available from: <https://wwwn.cdc.gov/nndss/conditions/lyme-disease/case-definition/2017/>.

52. Berglund J, Eitrem R, Ornstein K, Lindberg A, Ringnér Å, Elmrud H, et al. An Epidemiologic Study of Lyme Disease in Southern Sweden. *New England Journal of Medicine* (1995) 333(20):1319-24. doi: 10.1056/NEJM199511163332004.

53. Kullberg BJ, Berende A, Evers AW. Longer-Term Therapy for Symptoms Attributed to Lyme Disease. *N Engl J Med* (2016) 375(10):998. doi: 10.1056/NEJMc1608044.

54. Halperin JJ, Shapiro ED, Logigian E, Belman AL, Dotevall L, Wormser GP, et al. Practice Parameter: Treatment of Nervous System Lyme Disease (an Evidence-Based Review): Report of the Quality Standards Subcommittee of the American Academy of Neurology. *Neurology* (2007) 69(1):91-102. doi: 10.1212/01.wnl.0000265517.66976.28.

55. Halperin JJ. Nervous System Lyme Disease: Diagnosis and Treatment. *Rev Neurol Dis* (2009) 6(1):4-12.

56. Berende A, ter Hofstede HJ, Vos FJ, van Middendorp H, Vogelaar ML, Tromp M, et al. Randomized Trial of Longer-Term Therapy for Symptoms Attributed to Lyme Disease. *New England Journal of Medicine* (2016) 374(13):1209-20. doi: doi: 10.1056/NEJMoa1505425.

57. Brorson O, Brorson SH. An in Vitro Study of the Susceptibility of Mobile and Cystic Forms of *Borrelia Burgdorferi* to Hydroxychloroquine. *Int Microbiol* (2002) 5(1):25-31. doi: 10.1007/s10123-002-0055-2.

58. Brorson O, Brorson SH. An in Vitro Study of the Susceptibility of Mobile and Cystic Forms of *Borrelia Burgdorferi* to Tinidazole. *Int Microbiol* (2004) 7(2):139-42.

59. Brorson O, Brorson SH. An in Vitro Study of the Activity of Telithromycin against Mobile and Cystic Forms of *Borrelia Afzelii*. *Infection* (2006) 34(1):26-8. doi: 10.1007/s15010-006-4121-0.

60. Lantos PM, Auwaerter PG, Wormser GP. A Systematic Review of *Borrelia Burgdorferi* Morphologic Variants Does Not Support a Role in Chronic Lyme Disease. *Clin Infect Dis* (2014) 58(5):663-71. doi: 10.1093/cid/cit810.

61. Celik T, Celik U, Komur M, Tolunay O, Donmezer C, Yildizdas D. Treatment of Lyme Neuroborreliosis with Plasmapheresis. *J Clin Apher* (2016) 31(5):476-8. doi: 10.1002/jca.21430.

62. Patel R, Grogg KL, Edwards WD, Wright AJ, Schwenk NM. Death from Inappropriate Therapy for Lyme Disease. *Clin Infect Dis* (2000) 31(4):1107-9. doi: 10.1086/318138.

63. Eggers CH, Samuels DS. Molecular Evidence for a New Bacteriophage of *Borrelia Burgdorferi*. *Journal of Bacteriology* (1999) 181(23):7308-13.

64. Cameron DJ, Johnson LB, Maloney EL. Evidence Assessments and Guideline Recommendations in Lyme Disease: The Clinical Management of Known Tick Bites, *Erythema Migrans* Rashes and Persistent Disease. *Expert Rev Anti Infect Ther* (2014) 12:1103-35.

65. ILADS. The International Lyme and Associated Diseases Society. Evidence-Based Guidelines for the Management of Lyme Disease. (2004).

66. Halperin JJ. Chronic Lyme Disease: Misconceptions and Challenges for Patient Management. *Infect Drug Resist* (2015) 8:119-28. Epub 2015/06/02. doi: 10.2147/IDR.S66739.

67. Klempner MS, Hu LT, Evans J, Schmid CH, Johnson GM, Trevino RP, et al. Two Controlled Trials of Antibiotic Treatment in Patients with Persistent Symptoms and a History of Lyme Disease. *New England Journal of Medicine* (2001) 345(2):85-92.

## Suppl. Mat. 2: List of main research projects and products

The main research projects that were active in 2016 and 2017 are listed above (see table S2). They were obtained through the interview and the Tick and Tick-borne disease French group (<http://tmt.sfecologie.org/>). We also mentioned a short list of international project, which is by no means an exhaustive list. Projects were including because they had completely different approach than French projects or because they were heavily publicized. Finally we list different products that were available in France.

**Table 2S:** Main research projects addressing the fight against Lyme disease in France active in 2016 and 2017 in France. The project acronym are either the one given in the project or one we compiled in order to be able to refer to the project in the CK diagram.

| **Project acronym** | **Project name** | **Description** | **Main unit in charge** | **Period** |
| --- | --- | --- | --- | --- |
| **On ticks and tick-bites** | | | | |
| CiTIQUE | Citizens and ticks | The CiTIQUE project aims to involve citizen to better understand and manage the actual risks posed by tick-borne diseases. A first facet of the project has the development of the SIGNALEMENT TIQUE that allows a person to notify on a shared map where she or her domestic animal has been bit by a tick. The other facet of the program is the organization of citizen research internships accessible to all, during which participants can learn about and participate in the research on ticks and tick-borne disease. | INRAE Nancy | Since 2017 |
| CCEID & CLIMATICK | Projection and adaptation of tick threat in agricultural and forest landscape under climate change | The projects develop population dynamic to develop models for predicting the risks of emergence of tick-borne diseases, by integrating the effect of meteorology on the activity and dynamics of tick populations. These models are based on time series collected through the setting up of a network of observatories in metropolitan France. | UMR EPIA & ASTRE | Since 2014 |
| OSCAR | Simulation Tool for Mapping Acarological Risk in Agricultural Landscapes | The project works to create a cartography software capable of modelling the acarological risk according to agricultural landscapes. The project in parallel will investigate the link between landscape and the epidemiology of tick-borne disease. | UMR BIPAR | 2011-2016 |
| Xenobio-Tick | Sequencing the tick transcriptome for the development of new acaricides | Deep sequencing of the tick transcriptome in order to identify and characterize new neuroreceptor genes, as divergent as possible from those of insects, which will be used as tick-specific targets for the development of new acaricides. | UMR BIOEPAR | 2017-2021 |
| BioPuceTick |  | High throughput detection of pathogens vectorized by ticks (in France and Europe) | UMR BIPAR | Since 2010 |
| ESPEVEC | Historical and contemporary factors driving the evolution of host specialization in disease vectors | The aim is to understand the factors that favor host specialization in ticks and the consequences of this evolution for the transmission of associated pathogens. | UMR MIVEGEC | 2014-2017 |
| GENIRIC |  | Sequencing of the *Ixodes ricinus* genome, vector of multiple human and animal pathogens | UMR BIPAR | 2016-2022 |
| **On *Borrelia* and microbiota and co-infection in ticks** | | | | |
| OH!Ticks | One-Health approach to identify threat posed by tick-borne pathogens responsible of unexplained infectious syndrome in humans and animals | The OH!Ticks project investigates potential Lyme disease co-infections. The project’s goal is to detect and identify, in animals and humans, by high throughput sequencing, known tick-borne pathogens that are not usually detected with current diagnostic tools. The project then aims to investigate the epidemiological link between the presence of a pathogen and the clinical presentation of an infected patient. | UMR BIPAR | 2017-2020 |
| ENDOTICK |  | Emergence of pathogenicity in tick endosymbiotic communities, a comparative genomic approach of evolutionary transitions in the bacterial genus *Coxiella* | UMR MIVEGEC | 2014-2016 |
| KINETICKS |  | Network analysis and modeling to describe *I. ricinus* dynamics and associated pathogens | UMR BIPAR | 2017-2019 |
| **On knowledge, surveillance and diagnosis of Lyme disease** | | | | |
| LymeBarometre | Lyme knowledge of general population | Awareness and knowledge of Lyme Borreliosis and tick bite prevention among the general population in France | Santé Publique France | 2016 |
| DIABOLYC | Skin diagnosis of late Lyme borreliosis | The DIABOLYC project develops a proteomic- and PCR-based diagnose Lyme disease from biopsies of the skin. | Univ Strasbourg | 2017-2020 |
| DIALYMAS |  | Early diagnosis of Lyme borreliosis by proteomics | Univ Strasbourg | 2016-2020 |
| LymeSnap | Incidence of chronic erythema migrans in “Pays des Combrailles”: participatory study in the general population using remote declaration | The LymeSnap project aims to get a more accurate estimate of the incidence of human Lyme disease cases in the Pays de Combrailles, Puy-De-Dôme, France. The project relies on a smartphone application that allows users to take pictures of rashes. The pictures are then analyzed by infectologists, who provide the patient with follow-up. | CHU Clermont-Fd | 2016-2017 |
| **On vaccine and care** | | | | |
| LymeVac |  | Vaccine against Lyme borreliosis, with first development in dogs. | Univ Strasbourg, CNRS-IPHC | 2013-2017 |
| VacTix | Identification of tick molecules that contribute to tick-borne pathogens transmission and development of next generation vaccines against their transmission | The project aims at developing anti-tick vaccines for use in animals. One vaccine in development targets tick salivary proteins. | UMR BIPAR | 2014-2017 |
| ImmuneLyme Chronic | Immune response in CLD | Analysis of immune response by genomics and proteomics in CLD |  | 2016-2019 |
| Multi-specialty Lyme consultations |  | Multi-specialty Lyme consultations to allow the patient to see multiple relevant medical specialists in the place within a reduced timespan. | The Nancy CHRU | Since 2017 |

### Notable projects outside of France

- *NanoTrap Lyme Antigen Test:* Perhaps the most publicized project outside of France, the Ceres Nanoscience NanoTrap Lyme Antigen diagnostic test is based on western blot detection of *Borrelia* OspA (outer surface protein) detection from urine samples. As of early 2017, the tools are currently available for use only through Ceres NanoScience - <https://www.ceresnano.com/press-release-breakthrough> .
- *Bacteriophage project:* The Bacteriophage project is funded by Phelix Charisty company and is conducted at Leicester University and is hoping to develop bacteriophages as a diagnostic and treatment tool against Lyme disease. As of early 2017, the project was underway (<http://phelix.info/en/>).
- *Mice Against Tick:* Developed within the MIT Media Lab the project hopes to permanently immunizing mouse populations to block transmission by making and releasing mice that produce protective mouse antibodies from birth and pass immunity on to their pups. As of 2017, the project was still underway but was published subsequently (Buchthal et al 2019. <https://doi.org/10.1098/rstb.2018.0105>).
- *Rodent vaccine pellets:* US Biologics has developed vaccine-laced pellets against *Borrelia*. The vaccine has been tested. As of early 2017, the pellets were still under research (<https://usbiologic.com/>).

### Networks and products

- *Sentinelles*. Since 2009, the Réseau Sentinelles brings together medical providers and researchers in the surveillance of human cases of Lyme disease in France. It is managed by Santé Publique France and the UMR IPLESP.
- *PMSI – Medical program of information systems*. Database regarding automatic data collected on hospitalized patients. Managed by Santé Publique France. Since 2012.
- *Signalement TIQUE.* This application developed by INRA, DGS and ANSES allows a person to notify on a shared map where she or her domestic animal has been bit by a tick (launched in July 2017) - <https://www.citique.fr/signalement-tique/> .
- *FleaTickRisk* is a forecasting model proposed by Merial that uses meteorological data to predict ectoparasite activity in different climates. Weekly updated maps show the risk of infestation by ticks, fleas, mosquitoes and sandflies across Europe, to adapt pets' treatment to protect them from parasites (launched in 2019) - <https://www.fleatickrisk.com/fr> .
- *Anti-tick socks:* Developed by Labonal, these socks are built with an active ingredient that is said to resist multiple washes - <https://www.labonal.fr/fr/histoires-de-chaussettes/85-chaussette-anti-tiques-effet-permanent> .
- *VLA15 vaccine:* Valneva developed a human Lyme disease vaccine that will during 2017 enter phase 1 trials. The vaccine targets six *Borrelia* OspA (outer surface protein) serotypes - <https://valneva.com/research-development/lyme-disease/> .
